# Reporting practices and sourcing of food images in fMRI research: implications for reproducibility

**DOI:** 10.3389/fnhum.2026.1884065

**Published:** 2026-07-22

**Authors:** Andy Wai Kan Yeung

**Affiliations:** Oral and Maxillofacial Radiology, Applied Oral Sciences and Community Dental Care, Faculty of Dentistry, The University of Hong Kong, Hong Kong, China

**Keywords:** database, fMRI, food database, food image, food photo, reporting standard, visual food cue

## Abstract

The fMRI literature has accumulated many studies that investigated the neural correlates of viewing food images. However, there has been no literature survey to provide an overall understanding of which food image datasets were used by these studies. To address this gap, a meta-research was conducted to identify if the fMRI studies used food images from established datasets and exactly from which datasets. The online database of Web of Science Core Collection (WOSCC) was queried to identify relevant papers. After screening, the analysis consisted of data manually extracted from 490 papers published during 2001–2024. Approximately 53.7% of the 490 analyzed papers did not specify their source of food images. About 29.8% of papers referred to published datasets for their food images, whereas some papers utilized a combination of online stock photo and in-house library. The most frequently cited food image datasets were IAPS and Blechert et al. (2014). The popularity of IAPS seemed to be declining since the mid-2010s in favor of newer datasets such as Blechert et al. (2014), Charbonnier et al. (2016), and Blechert et al. (2019). Some datasets are no longer accessible or have changed their websites. The findings highlight substantial heterogeneity and underreporting of visual stimulus materials, which may contribute to variability in observed neural responses and limit cross-study comparability in fMRI research.

## 1 Introduction

The fMRI literature has accumulated many studies that investigated the neural correlates of taste and food stimuli. As a recent meta-evaluation pointed out, tens of meta-analyses have been published to include original studies that used numerous forms of food stimuli, ranging from food images, tastants (taste solutions), food odors, real food items, to mental imagery (Yeung et al., 2019). Meta-analyses showed that food images induced the most extensive and robust activation across the brain especially the visual cortex (van der Laan et al., 2011), whereas taste and smell stimuli induced stronger activation in the insula, the primary taste cortex (Huerta et al., 2014). It demonstrated the complexity and multi-faceted nature of the cerebral processing of food. In terms of tastants, the fMRI literature has been relatively standardized in the selection of chemicals to provide the five basic tastes, with popular examples being sucrose for sweetness, citric acid for sourness, quinine for bitterness and sodium chloride for saltiness (Yeung et al., 2017; Yeung et al., 2018). However, the situation for food images seemed to be different. The descriptions of food images seemed to varied, with one study describing the stimuli as “roasted chicken, hamburger, chocolate cake, strawberries, etc.” to another study with a simple description as “food” [see (Huerta et al., 2014) for the examples]. Since multiple food images may be used in a single study, authors may not list out everything in the article for simplicity or due to page restrictions. Hence, a viable alternative may be to disclose the sources of food images.

One popular picture database is the International Affective Picture System (IAPS), developed by the National Institute of Mental Health (NIMH) Center for Emotion and Attention at University of Florida. It seems that the release of IAPS was officially introduced in some technical documents instead of a journal publication (Lang et al., 1997). IAPS includes over 1,000 color photos that depict a wide variety of events and objects (including food images), and were rated by large standardized groups for evoked feelings of arousal, dominance, and pleasure (Bradley and Lang, 2017). Notwithstanding, IAPS was not specifically designed for food studies, such as the food items presented may have limitations. Food image datasets have since been developed, such as Food-pics (Blechert et al., 2014). It has a broader coverage of food items, such as vegetables versus meat versus fruits, high versus low-calorie food, sweet versus salty food, and whole versus processed food.

Differences in visual stimulus sets may influence neural activation patterns, particularly in regions such as the visual cortex and reward-related circuits, making transparent reporting of stimulus materials critical for interpreting and comparing fMRI findings (van der Laan et al., 2011; Arsenault et al., 2013). It was largely unclear if food studies have used food images from established datasets and exactly from which datasets. This could be particularly relevant to fMRI studies for which small methodological differences may lead to significantly different results (Carp, 2012; Botvinik-Nezer et al., 2020). Given this context, the purpose of this meta-research study is to characterize reporting practices and sourcing of food images in fMRI studies, and to evaluate their implications for reproducibility and comparability of neuroimaging findings.

## 2 Materials and methods

**Figure 1:**
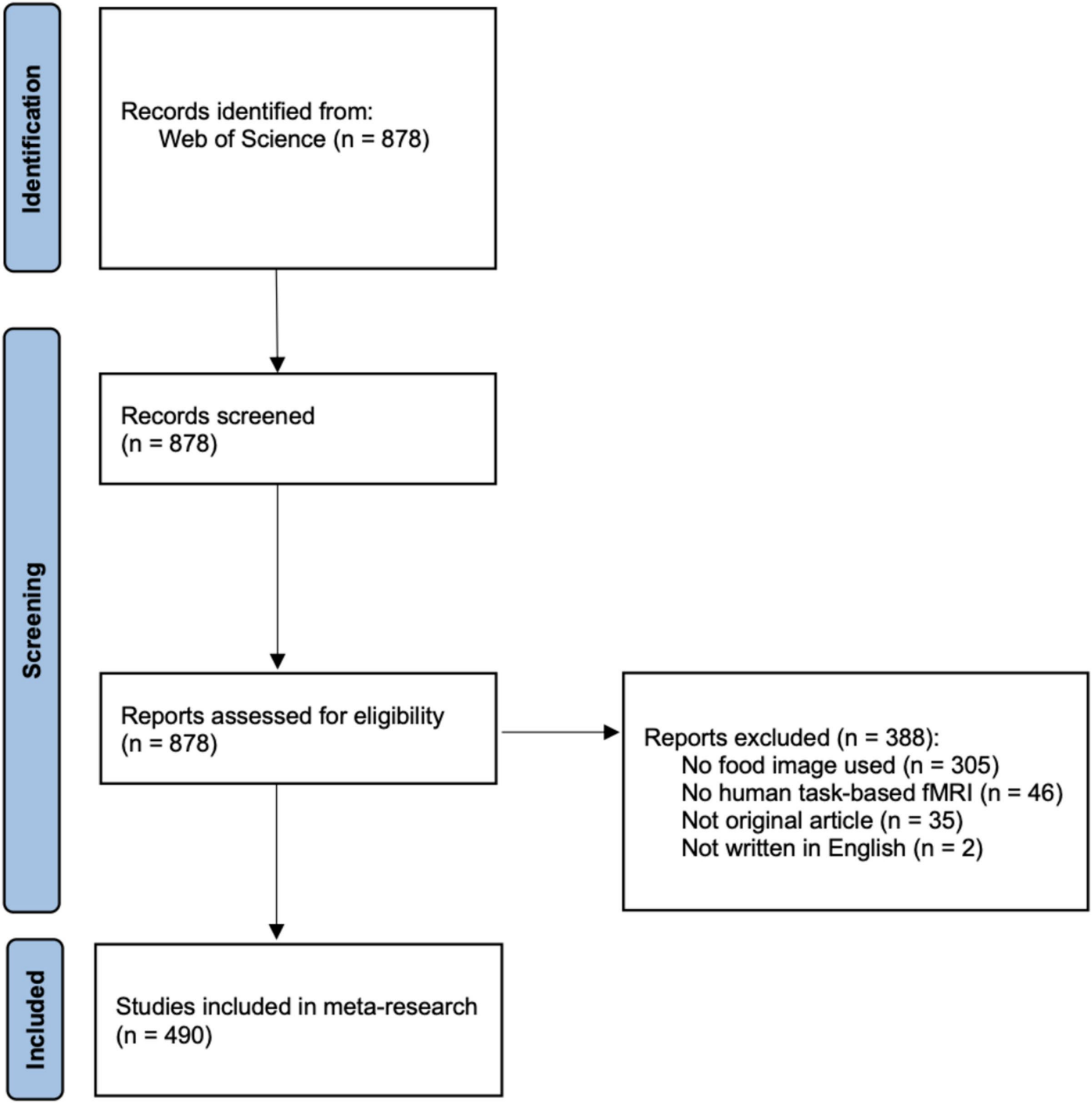
PRISMA-style flow diagram that illustrates the literature screening process.

The Web of Science Core Collection (WOSCC) online database was queried on 27 September 2024. The title and abstract fields were searched with the following search string: (fMRI OR “functional magnetic resonance imaging” OR “functional MRI”) AND (picture OR pictures OR image OR images OR visual) AND (food*). Papers were only included if they were labelled as articles. There were no other limitations placed on the search, such as the publication timespan or publication type. The search yielded 878 papers from WOSCC. Among these 878 papers, a total of 388 papers were excluded due to the following reasons: no food images used (*n* = 305), no human task-based fMRI performed (*n* = 46), not original article (*n* = 35), and non-English papers (*n* = 2) (Figure 1). For the remaining 490 papers that entered the analysis, the sources of food images and number of food images involved were manually extracted. In particular, the sources of food images were coded as follows: (1) unspecified; (2) online stock photo; (3) in-house library; (4) published datasets. Published datasets were defined as image sets that were formally described in publicly accessible sources, including peer-reviewed articles, technical reports, or other citable documents, and were intended for reuse in experimental research.

Statistical tests were performed with SPSS Version 28.0 (IBM, NY, United States). Linear regressions were conducted to reveal if there were significant annual trends in the ratio of papers with unspecified food image source, sources from published datasets, and food image from other sources (i.e., online stock photo and/or in-house library), respectively. Results were considered significant if *p* < 0.05.

## 3 Results

**Figure 2:**
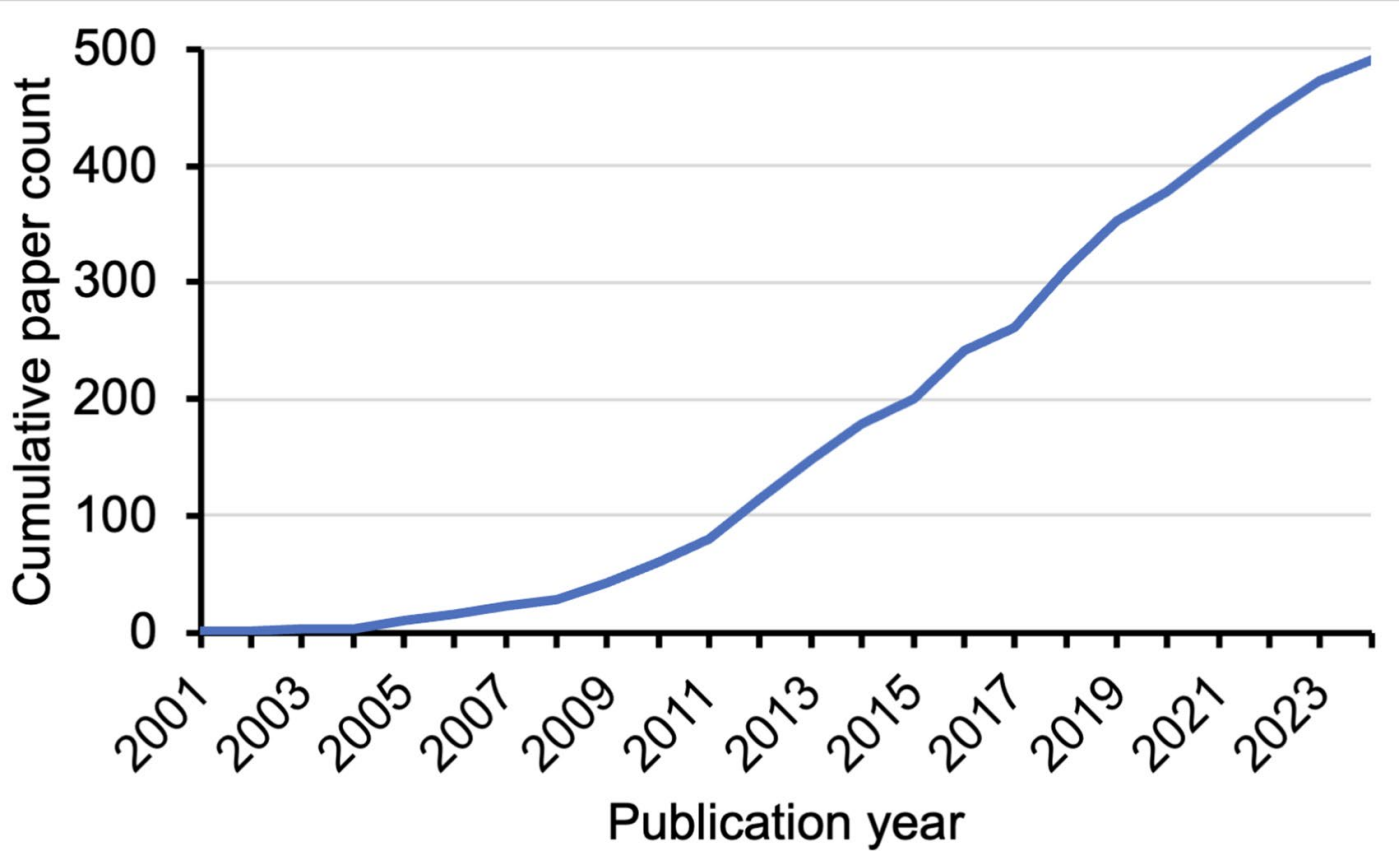
Cumulative publication count of fMRI studies that used food images.

The coded data sheet was provided as the Supplementary Data File S1. The first included paper was published in year 2001. The dataset included only 1 paper from 2001, no paper from 2002 and 2004, and 2 papers from 2003. From 2005 onwards, multiple papers were found in each year (Figure 2). Approximately 53.7% of the 490 papers did not specify their source of food images, whereas published datasets were cited in another 29.8% (Table 1). Some papers utilized a combination of published datasets, online stock photo, or in-house library.

**Table 1:** Overall breakdown of food image sources.

Sources	Number of papers (% of 490)
Unspecified	263 (53.7)
Published datasets	146 (29.8)
Online stock photo	32 (6.5)
In-house library	27 (5.5)
Online stock photo + published datasets	15 (3.1)
In-house library + published datasets	5 (1.0)
Online stock photo + in-house library	2 (0.4)

**Figure 3:**
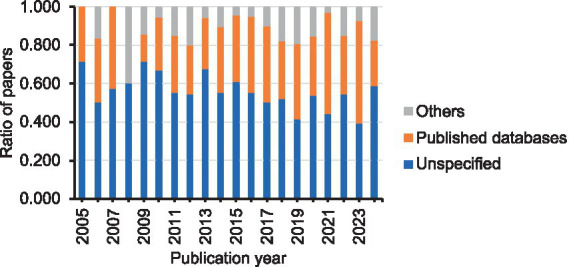
Annual trends of food image sources. Others meant only from online stock photo and/or in-house library.

Since the 3 papers published prior to 2005 either had unspecified food image source or used online stock photo, the annual trends of food image sources described in this paragraph were based on 487 papers published since 2005 (Figure 3). Linear regressions demonstrated that the ratio of papers with unspecified food image source had a 0.9% decrease per year over the period of 2005–2024 (*B* = −0.009, 95% CI: −0.015 to −0.003, *R* = −0.581, *p* = 0.007). Meanwhile, there were no significant temporal trends for the ratio of papers that involved published datasets as their food image sources (*B* = 0.009, 95% CI: −0.001 to 0.018, *R* = 0.423, *p* = 0.063) or from other categories (i.e., online stock photo and in-house library only, *B* = 0.000123, 95% CI: −0.008 to 0.008, *R* = 0.008, *p* = 0.974).

**Figure 4:**
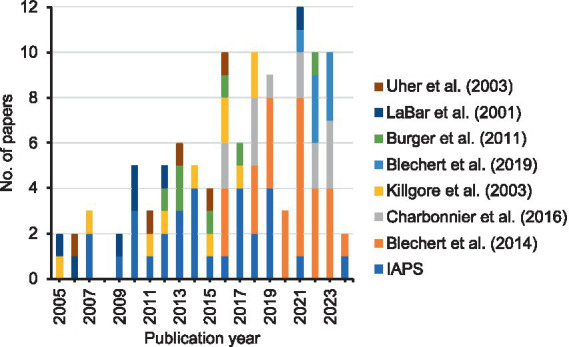
Annual trends of published datasets as food image sources.

Next, the published datasets were examined. Among the 166 papers that cited published datasets among their food image sources, 151 of them cited 1 dataset, 14 cited 2 datasets, 1 cited 3 datasets, and 1 cited 4 datasets. The most frequently cited food image datasets were IAPS (*n* = 30) and Blechert et al. (2014) (*n* = 29). Six other datasets were cited 5 times each (Table 2). The popularity of IAPS seemed to be declining since the mid-2010s in favor of newer datasets such as Blechert et al. (2014), Charbonnier et al. (2016), and Blechert et al. (2019) (Figure 4).

**Table 2:** Published datasets of food images that were cited at least 5 times by the analyzed literature set.

Datasets	Number of papers (% of 490)	Number of images/dataset information	Low-level visual features reported?
International affective picture system (IAPS)	30 (6.1)	>1,000 general affective pictures, including food images	Normative affective ratings reported; low-level food-specific values such as contrast/brightness/spatial frequency not clearly reported
Food-pics (Blechert et al., 2014)	29 (5.9)	568 food images	Yes: physical image characteristics including color composition, contrast, brightness, size, and complexity
F4H (Charbonnier et al., 2016)	13 (2.7)	286 initially created; final validated subset of 80 images	Standardized photographing protocol; subjective ratings reported, but low-level values such as contrast/brightness/spatial frequency not clearly reported
(Killgore et al., 2003)	11 (2.2)	Number not clearly specified	No per-stimulus low-level values clearly reported
Food-pics_extended (Blechert et al., 2019)	7 (1.4)	896 images	Extension of Food-pics; additional images and ratings reported
(Burger et al., 2011)	7 (1.4)	127 food images	Images standardized for contrast and brightness, but per-stimulus low-level values not clearly reported
(LaBar et al., 2001)	7 (1.4)	Number not clearly specified	Images formatted to same resolution/approximate size; no per-stimulus low-level values clearly reported
(Uher et al., 2003)	5 (1.0)	Number not clearly specified	No per-stimulus low-level values clearly reported

**Figure 5:**
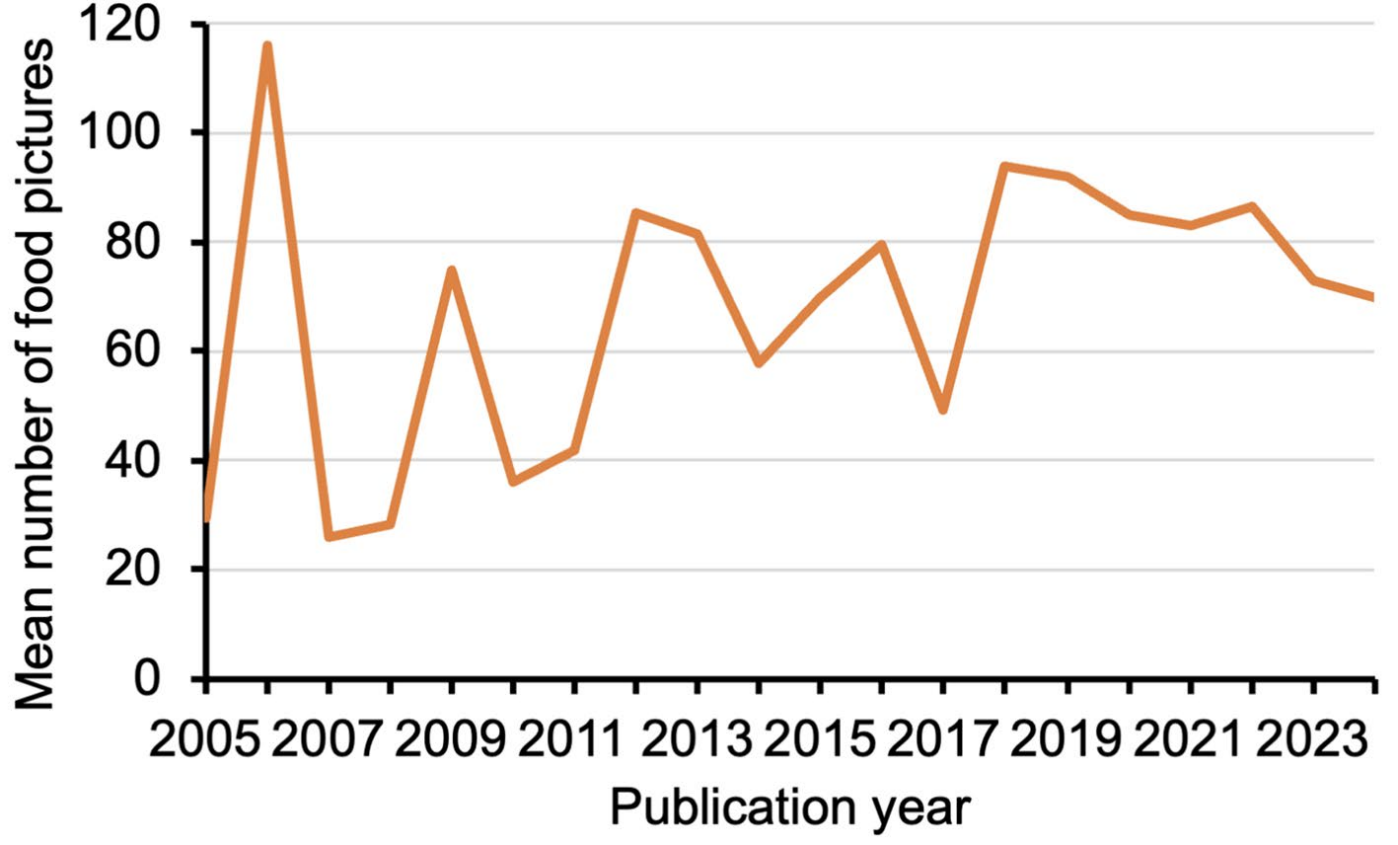
Mean number of food images used per study.

Finally, 281 of the 490 papers (57.3%) did not specify how many food images were used in their fMRI study. For papers that mentioned the exact numbers, the overall average number of food images used per study was 76.5. The mean number of food images used per study showed an increase of 1.9 per year, but the linear trend was not significant (*R* = 0.434, *p* = 0.056) (Figure 5).

## 4 Discussion

This literature survey has confirmed that IAPS was the most frequently used food image source for fMRI studies. However, its popularity faded in recent years in favor of newer datasets such as Blechert et al. (2014), Charbonnier et al. (2016), and Blechert et al. (2019).

IAPS is a validated and widely used picture database for researchers not limited to neuroimaging. However, readers may be less familiar with other frequently used datasets listed in Table 2 of the current report, hence they are further elaborated below.

First, LaBar et al. (2001) was an fMRI study. They collected color photos of food and tool from commercially available CD-ROMs and formatted them to the same resolution and approximate size. A subset of the images was Gaussian-blurred to become unidentifiable to serve as a low-level baseline. There was not a particular description on the number and content of food images included.

Next, Uher et al. (2003) was an fMRI study. The color photos of food were produced by the photography department of the Maudsley Hospital, which included sweet and salty foods presented on plates. Several food items were mentioned as examples, such as bread, cake, chocolate, pasta, pie, pizza, and rice. There was not a particular description on the number of food images produced.

Killgore et al. (2003) was another fMRI study. They obtained color photos of high-calorie and low-calorie food from commercial stock photo websites. The former group included barbequed chicken, chocolate chip cookies, cheeseburgers, French fries, hot dogs, ice cream sundaes, pasta with meat sauce, and strawberry shortcake. The latter group included fruits, garden salads, vegetables, and whole-grain cereals. There was not a particular description on the number of food images involved.

Burger et al. (2011) was a non-fMRI, cross-sectional survey study. Initially, 600 color photos of food were collected from lab personnel, online stock photos, IAPS, and previous fMRI research. After screening and exclusion, the dataset consisted of 127 food images that spanned across different categories. During presentation to the participants, some foods were presented twice with small portion size and large portion size (doubling the small portion). The images were standardized in terms of contrasts and brightness.

Blechert et al. (2014) was another non-fMRI study specifically aimed to establish a food image database called Food-pics. Color images were collected from a commercially available database, online stock photos, and produced by their lab. Photos were standardized on resolution, white background color, viewing distance, angle and simple figure-ground composition. Some foods were presented without dishware (such as fruits or hamburger), while others were presented with a plate or bowl (such as soup or fruit salad). A total of 568 food images were compiled. Food items were also presented in a single unit, multiple units, and as full meals. The following meta-data of the food images were provided: subjective normative ratings of valence, arousal, palatability, desire to eat, recognizability, and visual complexity; and objective data on macronutrients, energy density, and physical image characteristics (color composition, contrast, brightness, size, complexity).

Charbonnier et al. (2016) was another study aimed to establish a food image database called Full4Health Image Collection (F4H). It argued that the database created by Blechert et al. (2014) might have a few shortcomings arisen from collecting food images, pasting on a plain background, and adjusting the contrast and brightness. Subsequently, Charbonnier et al. (2016) created all food images by themselves under a standardized environment, to resemble the viewing of food presented on a 17 cm-diameter white plate on a light gray table during meal time. A total of 286 food images were initially created with this photographic protocol. After testing for recognizability by the general population in three European countries, the final subset consisted of 80 photos labelled with subjective ratings for liking, perceived calories and perceived healthiness. The original website listed in the study no longer works^1^ but the database can be accessed from a new website.^2^

Finally, Blechert et al. (2019) was an update to Blechert et al. (2014), and renamed the Food-pics database into Food-pics_extended. Several researchers that used the old Food-pics database have contributed by creating more food images in a similar style. By adding 328 new photos to the existing 568, the Food-pics_extended database now contains 896 photos. The original website listed in the study no longer works^3^ but the database can be accessed from a new website.^4^

Of course, there are other food image datasets in the literature but they are seldom or not used by the papers included in the current analysis. For instance, there is a database called FoodCast research image database (FRIDa) (Foroni et al., 2013). It contains 295 food images obtained from online stock photos and subsequently modified. One interesting aspect is that it contains a rotten food category that includes examples of rotten bananas, moldy Parmesan (cheese), and so on. This database was used by 2 of the 490 analyzed papers in the current study. Other food image datasets available in the literature included, but not limited to, Open Library of Affective Foods (OLAF) (Miccoli et al., 2014), Macronutrient Picture System (MaPS) (King et al., 2018), CROss-CUltural Food Image Database (CROCUFID) (Toet et al., 2019), and MacroPics (Fromm et al., 2021). They were not used by the papers analyzed in the current study.

On one hand, it may be intuitive to think that researchers should use identical food images from the exact datasets, so that results across studies can be more readily compared to each other. However, researchers often argue that foods, or diet, can vary widely due to differences in the cultural and geographic backgrounds. For instance, food items deemed to be familiar and palatable to the general population in European countries may not be well received by the population in the United States (Fromm et al., 2021). It could be too time-consuming and resource-demanding to take a series of original food images only to satisfy the need of a single research study. Therefore, some of the datasets mentioned above made use of online stock photos. Nowadays, generative artificial intelligence (genAI) has been advancing quickly and it may help researchers to produce realistic and customized food images. A noteworthy example is that Califano and Spence uploaded real food photos to ChatGPT-4 developed by OpenAI (San Francisco, United States), and asked it to generate prompts to reproduce the photos by DALL-E 3 also developed by OpenAI (Califano and Spence, 2024). Their results found that AI-generated food images closely resembled real photos, and, without disclosing the nature, participants preferred the former than the latter. In the future, customized and standardized food “photos” may be generated by AI without a photo shooting studio. When the technology matures, it should be particularly useful if researchers want to take “photos” of complicated dishes.

Nowadays, research community promotes openness and transparency, implying that researchers should report the materials and methods in details, so that fellow researchers can attempt to replicate, reproduce, or conduct follow-up studies. However, based on the current survey results, over half of the studies did not specify their food image sources or the number of food image used. The reporting can be more transparent in the future to allow for better replication or validation studies. Recently, an interesting neuroimaging meta-analysis on Stroop task studies has been published (Müller et al., 2024). In short, the original Stroop task requires participants to name the color of printed words that name incongruent colors, such as the word “blue” printed in yellow ink. The meta-analysis found that some studies tested a combination of emotional picture with word instead of color with word. Besides, the contrasting condition could be either congruent or neutral, with or without additional cognitive demands. All these different categories have led to different meta-analytic results. In a similar sense, fMRI studies that use food images as stimuli should also consider to report more methodological details particularly on the source and number of food images. An even better practice is to make the image set available to researchers in open access.

A related issue is the balance between reproducibility and stimulus variety. Using identical image sets across fMRI studies may improve direct comparability, but a restricted or homogeneous stimulus set may also limit generalizability and may bias findings toward the specific visual properties of those images. In the current survey, among studies that reported the number of food images used, the mean was 76.5 images per study. Moreover, low-level visual properties were inconsistently available across commonly used datasets. For example, Food-pics reported objective image characteristics such as color composition, contrast, brightness, size, and complexity, whereas several other frequently cited sources mainly reported affective/appetitive ratings or described image standardization without providing per-stimulus low-level values. Because low-level visual features can influence activity in visual and reward-related brain regions (Huffman and Stark, 2017), future fMRI studies using food images should report not only the source and number of stimuli, but also whether contrast, brightness, spatial frequency, size, and related image properties were controlled or made available.

Another issue identified in this survey related to the open accessibility of the food images. Several datasets elaborated above have changed their websites, so that the originally listed weblink no longer directs users to the referenced content. This issue is known as a reference rot, or more precisely, a link rot (Klein et al., 2014). An analysis of 3.5 million journal articles published in science, technology and medicine fields over the period of 1997–2012 has shown that approximately 20% of all articles suffered from reference rot, and 70–80% of articles that contained references to websites suffered from reference rot (Klein et al., 2014). This phenomenon was demonstrated in the current results with the examples of Food-pics and F4H databases, both of which have changed websites since the publication of their original study. Meanwhile, IAPS is no longer accessible from the website of University of Florida. Actively maintain the accessibility of the food image databases could be a resource demanding task which may possibly make people wonder who should take up this responsibility or whether it is beneficial to do so especially if it is not incentivized.

This study has some limitations. First, only fMRI studies were analyzed. The literature shares of the mentioned food image datasets could be very different if a different literature set would be analyzed. Second, the reported literature shares could be underestimated, as only explicit references to the published datasets were counted. Unclear or doubtful mentions were all conservatively treated as being unspecific in the current study, such as lines that said “the study paradigm closely followed Study X,” “we used the same task as Study X,” and so on. Third, the number of food images involved in a study only accounted for the food item itself but not for textual differences. For instance, a study that investigated food technologies for eggs used a photo of a package of eggs with the text labels of “caged hens,” “confined hens,” or “free-range hens,” with different price tags (Cherry et al., 2015). In the current analysis, this was treated as one food image involved. Moreover, only WOSCC was used as the literature source. It was used because it provided broad multidisciplinary coverage, standardized citation metadata, and reliable tools for longitudinal meta-research analyses. It has also been widely used in meta-research and bibliometric studies because of its structured citation records and relatively stringent journal indexing (Li et al., 2018). Readers should note that restricting to WOSCC might omit studies indexed only in other databases such as Scopus. Besides, data extraction was performed by a single reviewer without independent verification.

Although differences in stimulus sets, such as familiarity with gustatory stimulus (Staszko et al., 2022), may plausibly affect neural activation patterns, the present study did not examine associations between specific datasets and fMRI outcomes. Future work linking stimulus characteristics to activation variability would be valuable.

## 5 Conclusion

Based on a literature survey of fMRI papers published during 2001–2024, approximately 53.7% of the 490 analyzed papers did not specify their source of food images. About 29.8% of papers referred to published datasets for their food images, whereas some papers utilized a combination of online stock photo and in-house library. The most frequently cited food image datasets were IAPS and Blechert et al. (2014). The popularity of IAPS seemed to be declining since the mid-2010s in favor of newer datasets such as Blechert et al. (2014), Charbonnier et al. (2016), and Blechert et al. (2019). Meanwhile, IAPS is no longer accessible from its official website. Future studies should report more details about the food images utilized, such as their source, which and how many images were used. Food image datasets should also be stored in easily retrievable websites that are maintained in the long term, as the current results highlighted the issue of link rot, with the websites listed in the journal articles are no longer working.

## Data Availability

The original contributions presented in the study are included in the article/Supplementary material, further inquiries can be directed to the corresponding author.

## Footnotes

1
http://nutritionalneuroscience.eu/

2 https://osf.io/cx7tp/ or https://doi.org/10.17605/OSF.IO/CX7TP

3
www.food-pics.sbg.ac.at

4
www.eat.sbg.ac.at/resources/food-pics
